# Genetic characterization of fall armyworm (*Spodoptera frugiperda*) in Ecuador and comparisons with regional populations identify likely migratory relationships

**DOI:** 10.1371/journal.pone.0222332

**Published:** 2019-09-19

**Authors:** Rodney N. Nagoshi, Benjamin Y. Nagoshi, Ernesto Cañarte, Bernardo Navarrete, Ramón Solórzano, Sandra Garcés-Carrera

**Affiliations:** 1 Center for Medical, Agricultural and Veterinary Entomology, United States Department of Agriculture-Agricultural Research Service, Gainesville, Florida, United States of America; 2 University of South Florida, Tampa, Florida, United States of America; 3 National Institute of Agriculture Research (INIAP), Quito, Ecuador; National Cheng Kung University, TAIWAN

## Abstract

Fall armyworm, *Spodoptera frugiperda* (J. E. Smith), is an important agricultural pest native to the Americas that has recently been introduced into the Eastern Hemisphere where it has spread rapidly through most of Africa and much of Asia. The long-term economic consequences of this invasion will depend on how the species and important subpopulations become distributed upon reaching equilibrium, which is expected to be influenced by a number of factors including climate, geography, agricultural practices, and seasonal winds, among others. Much of our understanding of fall armyworm movements have come from mapping genetically defined subpopulations in the Western Hemisphere, particularly in North America where annual long-distance migrations of thousands of kilometers have been documented and modeled. In contrast, fall armyworm mapping in much of the rest of the hemisphere is relatively incomplete, with the northern portion of South America particularly lacking despite its potential importance for understanding fall armyworm migration patterns. Here we describe the first genetic description of fall armyworm infesting corn in Ecuador, which lies near a likely migration conduit based on the location of regional trade winds. The results were compared with populations from corn habitats in select locations in the Caribbean and South America to investigate the possible migratory relationship between these populations and was further assessed with respect to prevailing wind patterns and the distribution of locations with climate favorable for fall armyworm population establishment and growth.

## Introduction

The noctuid moth *Spodoptera frugiperda* (J. E. Smith) (Lepidoptera: Noctuidae), commonly called fall armyworm, is native to the Western Hemisphere. It is the primary insect pest of corn in the southeastern United States, the Caribbean, and South America and can cause significant economic damage in several other crops including sorghum, millet, cotton, and rice [1].

Fall armyworm is a tropical pest not known to diapause and so unable to survive severe winters. In North America known winter populations are limited to southern Texas and southern Florida [2], yet infestations annually extend thousands of kilometers northward as far as Canada [3]. This is due to an extensive annual migration beginning in the spring that modeling shows depends on three factors: 1) Seasonal air transport systems that direct northward migration, 2) Extensive corn acreage along the migratory route that support high density fall armyworm populations, and 3) Climate conditions favorable to fall armyworm development in the infested regions [4]. These characteristics are a consequence of the capabilities exhibited by many *Spodoptera* species of wind-based long-distance flights at high altitude that are limited to nocturnal hours [5]. Continuous flights of several hundred kilometers per night are possible under optimal conditions, but suitable staging areas are needed along the migratory route for rest and feeding during the day [5]. The North American migration occurs over several generations, hence the need for extensive corn acreage to support high density populations.

Long distance migration of fall armyworm in other regions has not yet been demonstrated. Whole genome variation comparisons suggest fall armyworm is a single breeding population across the entire hemisphere, consistent with frequent and general migration [6, 7], though there are indications of regional genetic heterogeneity in South America [8]. We have found using methods that are less sensitive to low frequencies of genetic introgressions that fall armyworm can be subdivided into two geographically separated groups [9]. One is the “TX-type” that include populations in that overwinter in Texas (and migrate to central North America), Mexico, Trinidad and Tobago, and South America. The other is the “FL-type” found in Florida, which migrate to the eastern United States, and is present in most of the islands surveyed in the Caribbean [3, 10, 11]. This pattern has been consistently observed for over 10 years and so represents the equilibrium distribution of fall armyworm in the Western Hemisphere.

The broad host range exhibited by fall armyworm is in part due to the presence of two subpopulations that differ in their host plant preferences [12, 13]. Historically designated as host strains their phylogenetic relationship remains unclear. They are morphologically indistinguishable except for evidence of wing size differences between strains in South America [14, 15], and there have been reports of strain differences in female pheromone constituents, mating behaviors, and physiology [16–24]. However, fall armyworm exhibits substantial variability between geographical populations independent of strain differences that can make identifying strain diagnostic traits problematic [25].

The strains were originally identified by genetic marker differences between larval specimens collected from rice versus corn, which gave rise to the designation rice-strain (RS) and corn-strain (CS) [13]]. Subsequent studies showed a stronger preference of RS to pasture grasses and millet, with rice being more variable, while the CS group is preferentially found in corn, cotton, and sorghum [26, 27]. The correspondence between markers and host plant has been observed throughout the Western Hemisphere but is not absolute, with on average about 20% of larvae from corn expressing RS markers and sporadic observations of more substantial disagreements [28–30]. Despite this variability, genetic markers remain the method of choice for strain identification.

Most useful for our studies have been genetic markers derived from portions of the mitochondrial *Cytochrome Oxidase Subunit I* (*COI*) and the sex-linked *Triosephosphate isomerase* (*Tpi*) genes [28, 31]. One segment of *COI* (COIA) includes a segment commonly used for DNA barcoding and which we typically use to confirm species identification [32]. A second segment, COIB, carries polymorphisms instrumental in distinguishing both strains and the FL-type/TX-type groupings [9, 33, 34]. The nuclear *Tpi* gene is also used as a marker of strain identity and may be more accurate than COIB [28]. Because of its location as a nuclear marker (as opposed to the mitochondrial *COI*), the *Tpi* marker can be used both in combination with *COI* and separately to explore the possibility of hybridization between strains [34, 35].

Fall armyworm recently invaded Africa, where it rapidly spread throughout the continent and has subsequently been found in India and southeastern Asia [36–39]. The potential economic consequences are significant and there is much interest in using the Western Hemisphere information to better understand how fall armyworm is moving in the Eastern Hemisphere and its eventual distribution at equilibrium. Both will be largely determined by seasonal wind patterns, regional and local environmental conditions, and the availability of host plants, particularly corn. With respect to climate, the situation in Africa will likely reflect that of South America more than North America.

The northern portion of South America has not been studied using our suite of markers yet represents a potentially important region for understanding fall armyworm migration patterns. The two American continents are linked by two obvious pathways for natural migration between the continents for flying insects. One is the land connection comprised of Central America and the Isthmus of Panama, and the other is the chain of islands in the Caribbean Sea known as the Greater and Lesser Antilles. Our previous studies indicated that large population movements in the Caribbean were unlikely with little mixing observed between fall armyworms from the two continents, but we have no data from Central America or Panama.

We are in the process of extensive surveys of fall armyworm in Ecuador, which is the closest location we have analyzed to date to the Isthmus of Panama and Central America. We present in this study the first genetic description of fall armyworm from cornfields in Ecuador, which was then compared with those from select corn-growing locations in the Caribbean and South America to investigate possible migratory relationships. The results were further assessed within the context of prevailing wind patterns during the collection period and the distribution of climate favorable for fall armyworm growth and establishment. We further demonstrate that COIB can differentiate fall armyworm from other *Spodoptera* species, eliminating the need to sequence COIA and thereby simplifying the genetic analysis.

## Results

### Species identification using COIB

**Fig 1:**
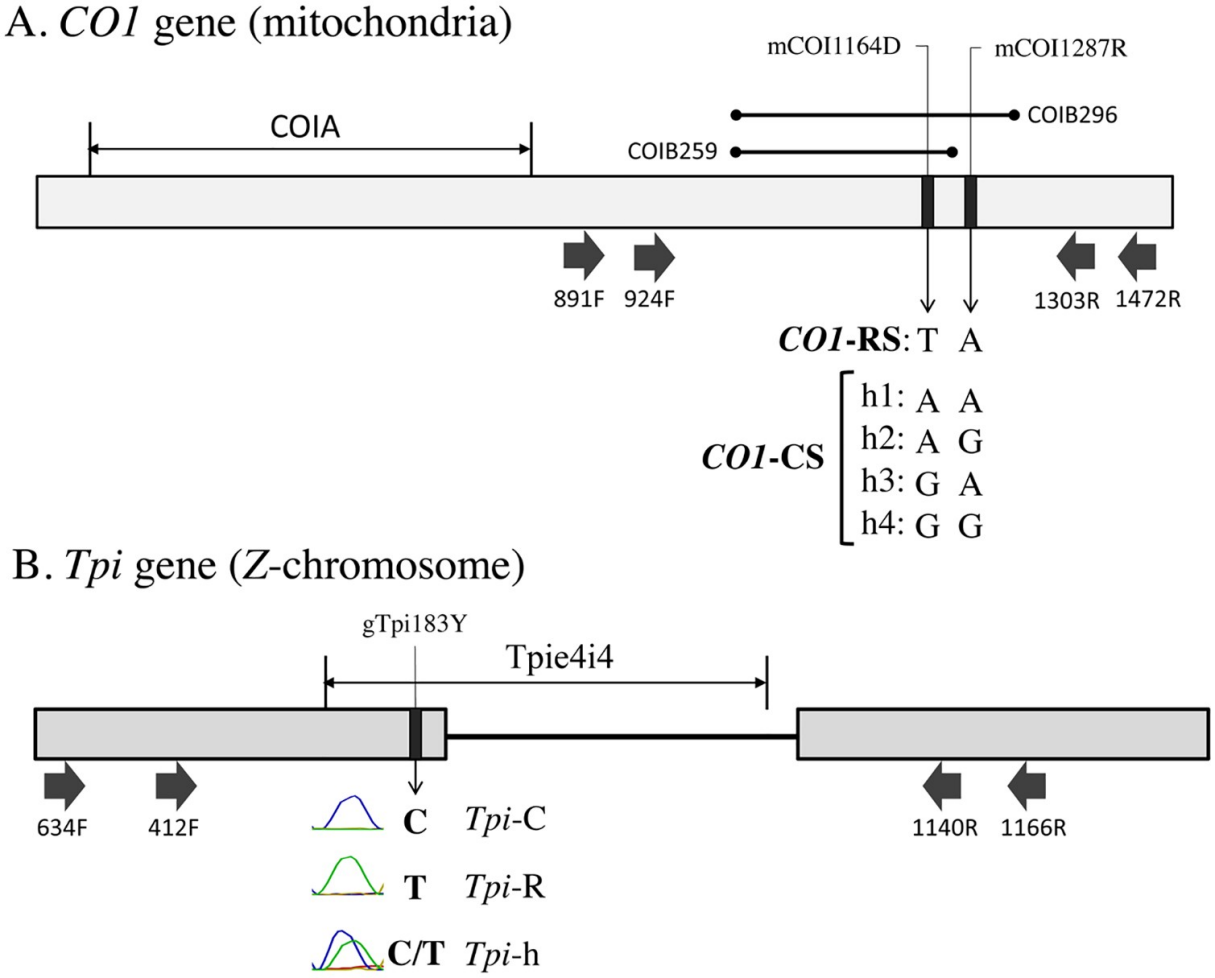
Diagrams of the COIB, *Tpi* gene segments. A. The COI gene showing the locations of the COIA segment frequently used for DNA barcoding and the COIB segments used in this study. The mCOI1164D site defines COI-based strain identity and together with polymorphisms at mCOI1287R describe the corn-strain h1-4 haplotypes. Black arrows indicate locations of primers used for PCR and DNA sequencing. B. Map of a section of the Z-linked Tpi gene that includes exon4 (fourth exon from the start of the coding region), exon5 and the intervening intron. The gTpi183Y site defines Tpi-based strain identity with the empirically observed sequence chromatographs shown indicating the C, T, C/T heterozygote forms found in this study. The Tpie4i4 contains a portion of exon4 and intron4.

The mitochondrial *COI* and nuclear *Tpi* genes contain polymorphisms useful for the analysis of fall armyworm populations (Fig 1). The 5’ segment of the *COI* locus, COIA, contains the barcode region capable of species and host strain identification [32], while the more 3’ segment, COIB, produce haplotypes that also identify strain and differentiate between two geographically separated populations [9]. To streamline the analysis by *COI* we tested whether COIB could substitute for COIA for species identification.

**Fig 2:**
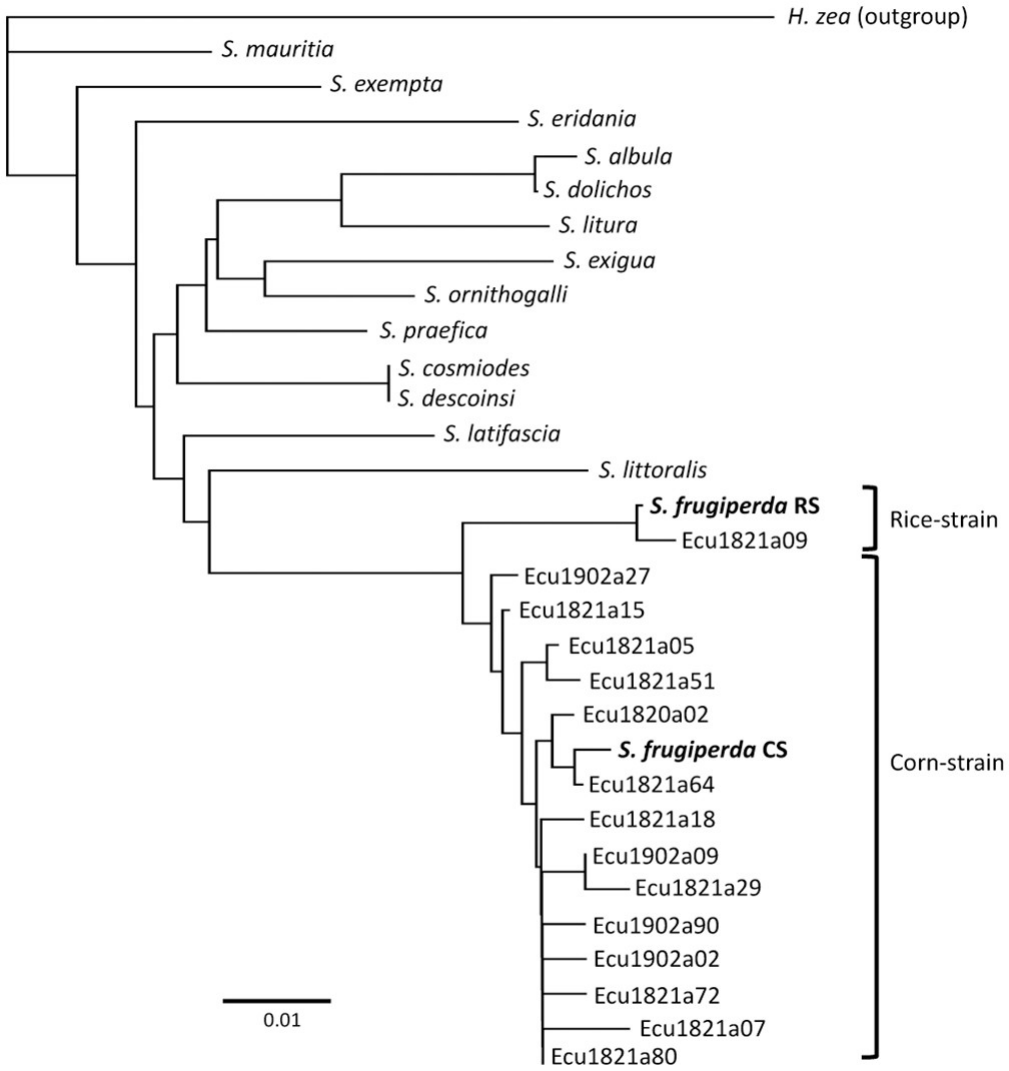
Phylogenetic tree inferred by using neighbor-joining method and Tamura-Nei model [40] with no outgroup. The sequences for the different Spodoptera species were obtained from GenBank. Corn-strain (*S*. *frugiperda* CS) and rice-strain (*S*.*frugiperda* RS) are consensus reference sequences (S1 Fig).

Sequence information from the COIB region were obtained for 13 *Spodoptera* species from GenBank. These were found to share a 259-bp COIB segment (designated COIB259). Despite the short length this sequence was sufficient to distinguish fall armyworm of both strains from the other *Spodoptera* species by Neighbor-joining analysis (Fig 2). *Spodoptera cosmiodes* (Walker) and *S*. *descoinsi* ((Lalanne-Cassou & Silvain) were identical in their COIB259 sequence, while *S*. *albula* (Walker) and *S*. *dolichos* (F.) appear to be closely related. A total of 143 specimens from Manabi, Ecuador were analyzed by *COI* sequencing with 22 COIB259 haplotypes observed. One Ecuador haplotype clustered with the consensus rice-strain COIB259 while the remainders were most similar to the consensus corn-strain haplotype (Fig 2).

### Strain-identity based on specific *COI* and *Tpi* polymorphisms

**Fig 3:**
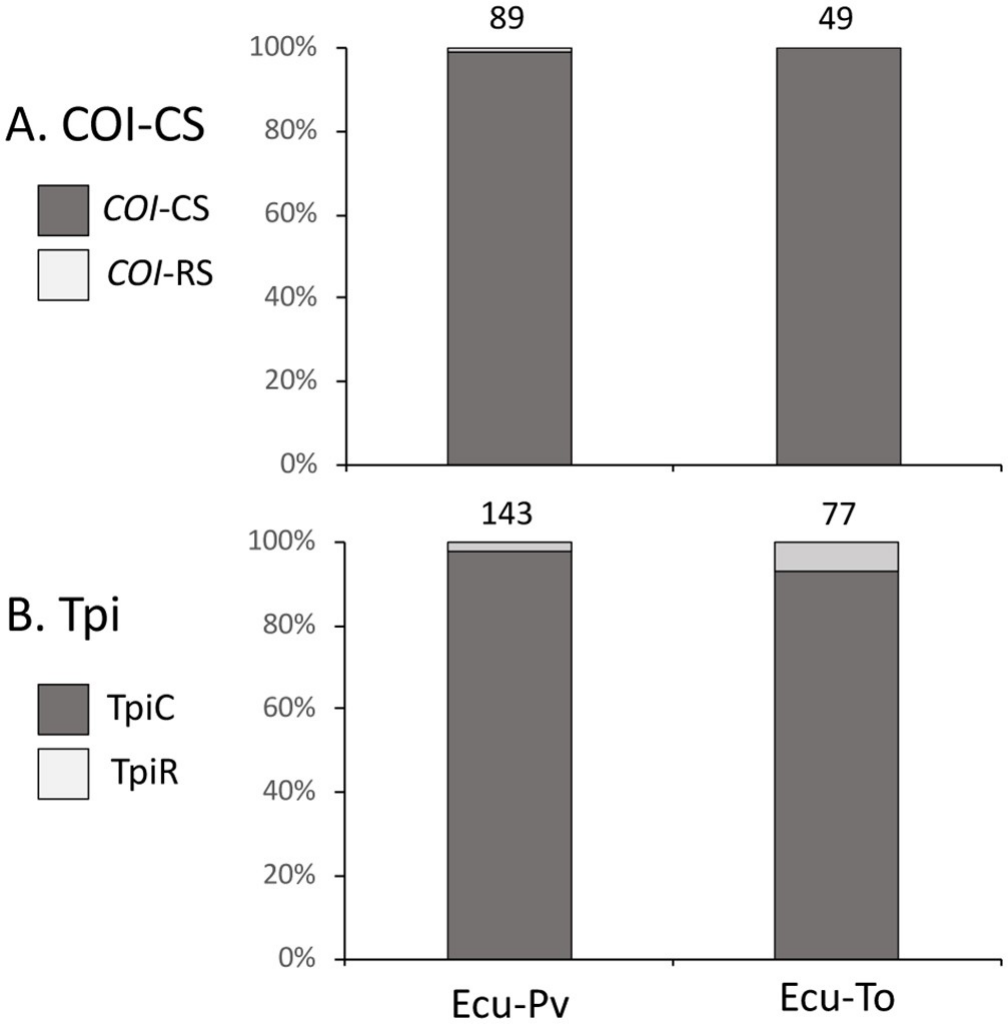
Comparisons of *COI* and *Tpi* haplotype frequencies of fall armyworm in Ecuador. A. Frequency of the *COI* strain diagnostic markers based on analysis of the COIB segment. Number of specimens indicated above columns. B. Frequency of the *Tpi* strain diagnostic markers from the TpiE4 exon segment. Estimated number of chromosomes tested indicated above columns.

The two strains can also be distinguished by polymorphic sites in the *COI* and *Tpi* genes (Fig 1). The site COIB_1164_ is strain-specific with a T_1164_ indicating rice-strain (*COI*-RS) and an A_1164_ or G_1164_ diagnostic of the corn-strain (*COI*-CS). The results from this single polymorphic site were consistent with the phylogenetic comparisons as the single Ecuador specimen clustering as *COI*-RS expressed T_1164_, while the remaining Ecuador haplotypes clustering with the consensus *COI*-CS sequence expressed either A_1164_ or G_1164_. Overall, the *COI*-CS haplotype predominated at all locations as expected since collections were limited to cornfields (Fig 3A).

The *COI* strain data were confirmed by the *Tpi* marker, where strain identification is defined by the gTpi183Y polymorphism in exon4 (Fig 3B). The Z-chromosome-linked *Tpi* gene is present in two copies in males (ZZ) and one copy in females (ZW), which means that a portion of the larval collections, which were not sexed, have the potential to be heterozygous for *Tpi*. As a result, three gTpi183Y sequencing chromatographs are observed that derive from homozygosity (ZZ) or hemizygosity (ZW) for C_183_ (TpiC or corn-strain), T_183_ (TpiR or rice-strain), or the presence of both C and T (TpiH or hybrid). The number of TpiC and TpiR chromosomes were estimated and both Ecuador locations were over 95% TpiC (Fig 3B).

### *COI*-h haplotype proportions

**Fig 4:**
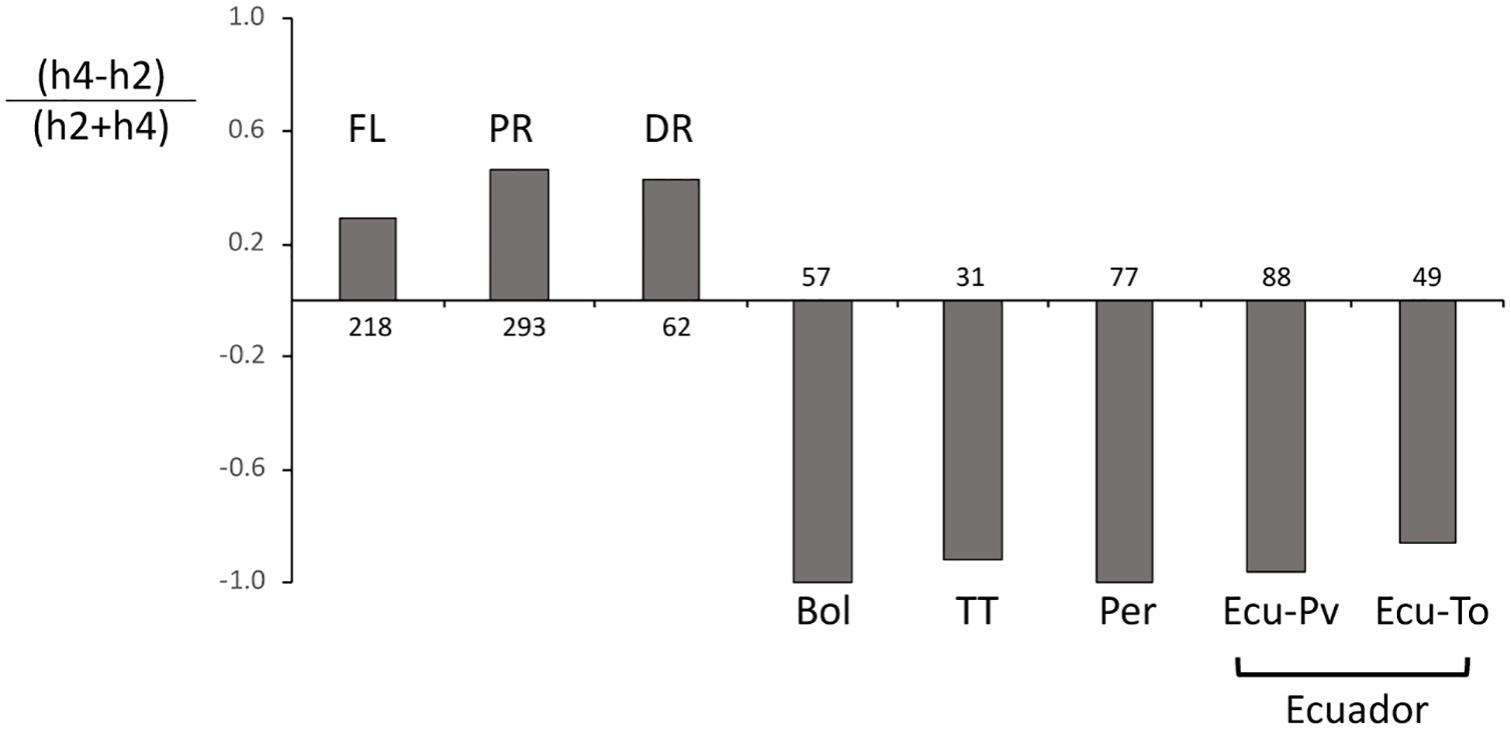
Frequency distributions of the COIB h-haplotypes, h1-4, for Ecuador and select locations in the Western Hemisphere. Numbers indicate the number of *COI*-CS specimens. Data for Florida (FL), Puerto Rico (PR), Dominican Republic (DR), Bolivia (Bol), Trinidad-Tobago (TT) and Peru (Per) were from Nagoshi et al. (2016) [33]. Ecuador specimens are from Portoviejo (Ecu-Pv) and Tosagua (Ecu-To).

Near the COIB_1164_ strain-diagnostic marker is a polymorphic site, COIB_1287_, that is partially strain-specific. The *COI*-RS T_1164_ variant is always associated with A_1287_, while the corn-strain A_1164_ or G_1164_ polymorphisms can be found with either A_1287_ or G_1287_. As a result, *COI*-RS is identified by the combination T_1164_A_1287_ while *COI*-CS includes four variants designated the *COI*-h haplotypes, A_1164_A_1287_ (h1), A_1164_G_1287_ (h2), G_1164_A_1287_ (h3), and G_1164_G_1287_ (h4) (Fig 1A). The relative proportions of h2 and h4 as measured by the metric (h4-h2)/(h2+h4) vary with location in such a way that they subdivide fall armyworm populations in the Western Hemisphere into two groups (FL-type and TX-type) [33]. Examination of specimens from Ecuador were of the TX-type, consistent with locations in South America and different from that found in the Greater Antilles and Florida (Fig 4).

### Haplotype network comparisons of *Tpi* variation

**Fig 5:**
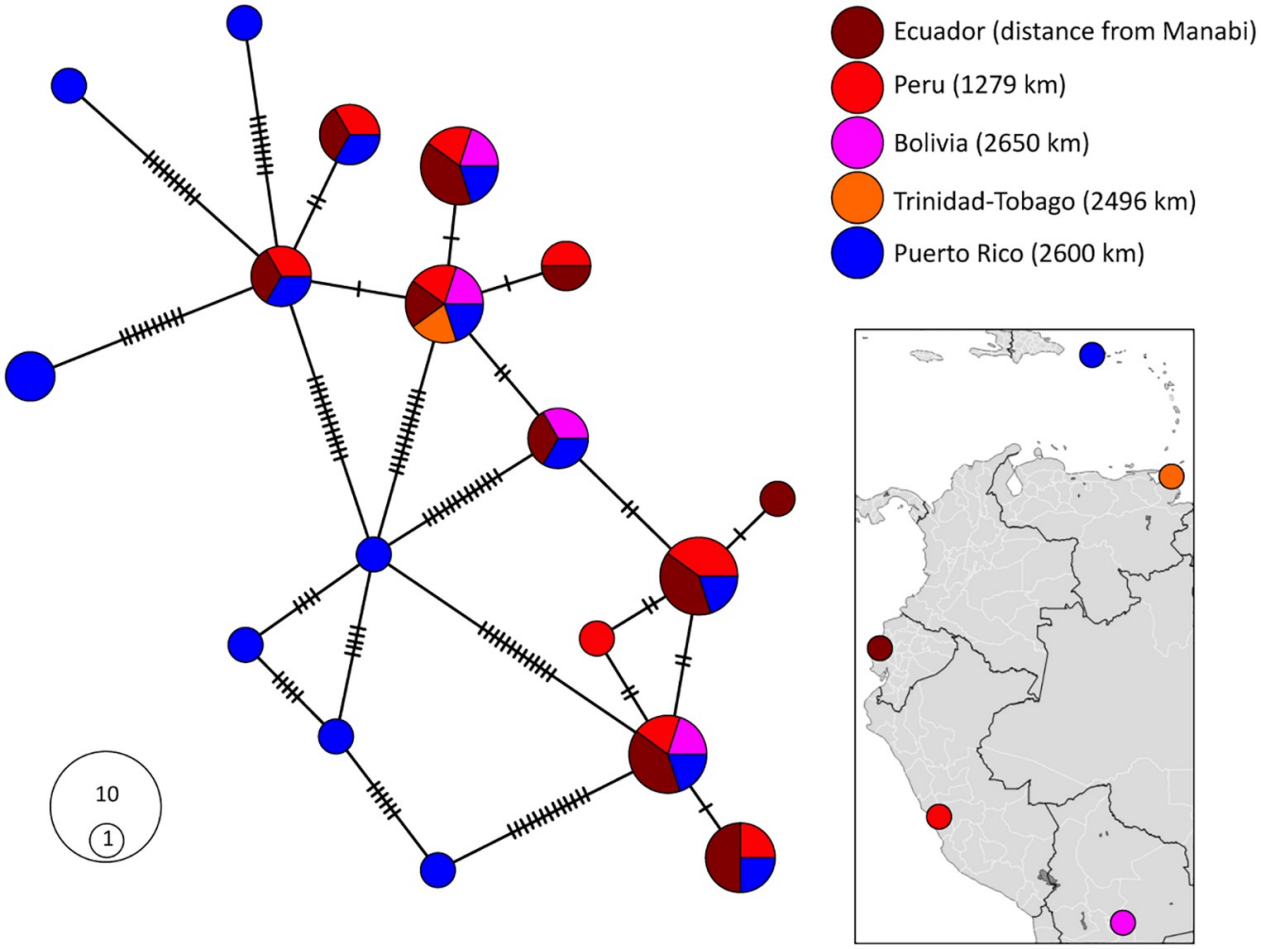
Haplotype network analysis using the Tpie4i4 segment of the *Tpi* gene illustrating the genetic differences between the haplotypes found in Ecuador, Peru, Bolivia, Trinidad-Tobago, and Puerto Rico. Kilometers in parenthesis indicate distance from Manabi, Eucador. Circle area proportional to number of haplotypes.

Network analysis was used to compare the genetic variation in the *Tpi* gene of Ecuador fall armyworm with those from four regional collection sites. The Tpie4i4 haplotypes identified from Ecuador were similar in sequence to each other, with no more than two mutations difference between one haplotype and the most closely related variant (Fig 5). There was considerable overlap in the haplotypes found in Bolivia, Peru, and Trinidad-Tobago with those in Ecuador and with each other, again with little variation between them. This was in marked contrast with the collections from Puerto Rico. Seven haplotypes unique to Puerto Rico were identified that each differed by at least eight mutations from those at the other locations. These results suggest that the Puerto Rico population is effectively isolated from the other locations and is in the process of genetic divergence. In contrast, there appears to be significant interactions between the fall armyworms in Ecuador, Peru, Bolivia, and Trinidad and Tobago despite the thousands of kilometers that separate each of these locations.

## Discussion

**Fig 6:**
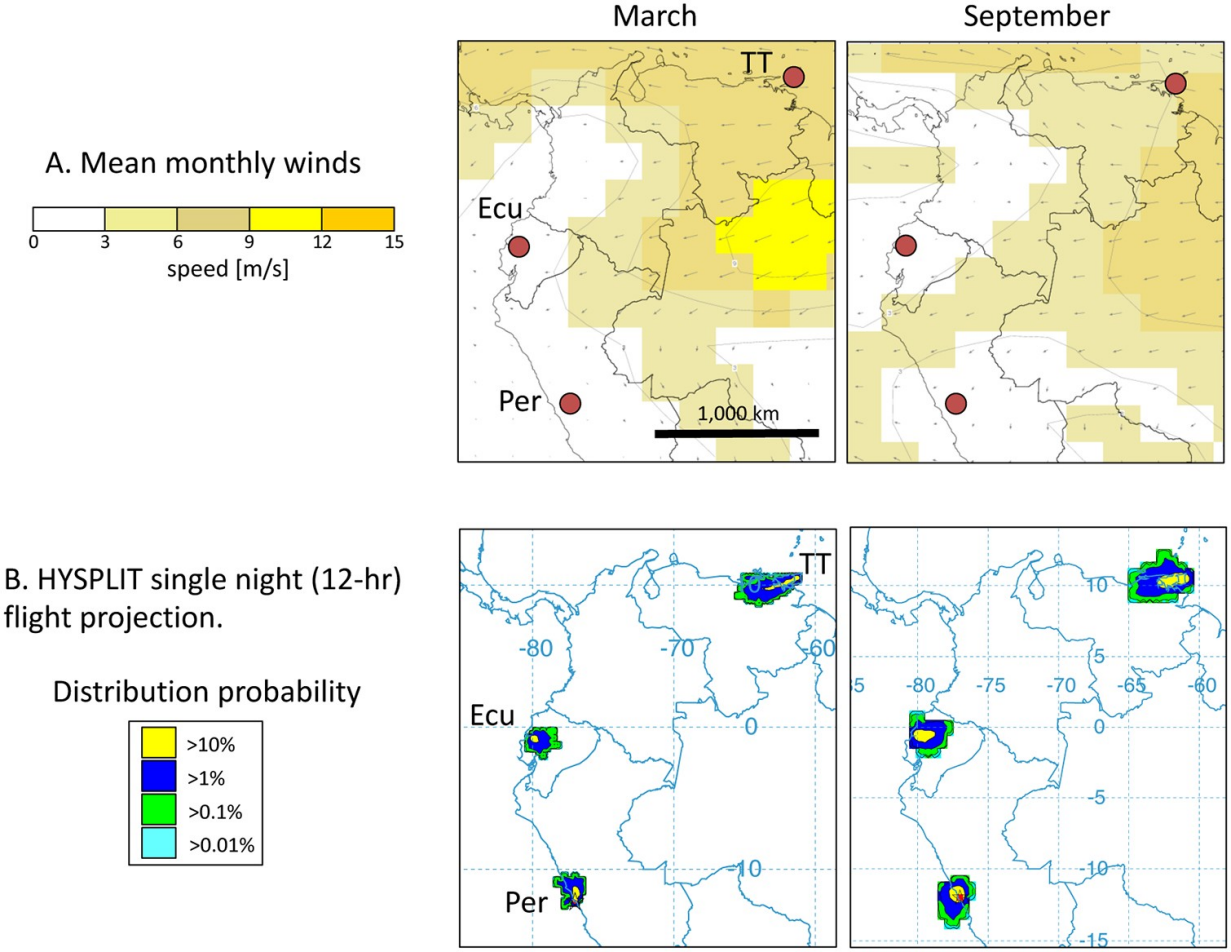
Maps indicating wind conditions in Ecuador and surrounding regions. **A. Map of vector mean winds Ecuador and surrounding areas**. Climatology of monthly vector mean winds from March and September at the 925-mb pressure-height from NCEP-NCAR Reanalysis data using the 1981–2010 base period. TT, Trinidad-Tobago; Ecu, Ecuador; Per, Peru. B. HYSPLIT forward projections of 12-h overnight trajectories at 500 m AGL based on historical wind data. Average monthly trajectories for selected months are displayed as a frequency distribution from Ecuador (Ecu: -0.78, -80.22), Peru (Per: -12.08, -76.93), Trinidad and Tobago (TT: 10.65, -61.43).

The characterization of fall armyworm from Ecuador provides an important data point from a region in the Western Hemisphere where fall armyworm has not been studied extensively with our suite of molecular markers. Ecuador is of particular interest because of its location relative to potential intercontinental migration pathways and air transport systems that are likely driving fall armyworm migration in South America. The South American Low Level Jet is the predominant lower altitude wind system in the region, forming an easterly trade wind from the equatorial Atlantic that is deflected sharply south-southeastward by the higher elevations of the Andes Mountain Range [41]. This occurs just south of Ecuador and most likely directs migration of fall armyworm populations to the southern and eastern portions of the continent. With respect to the survey locations, these observations suggest that populations in Trinidad and Tobago could contribute to fall armyworm in Ecuador, which in turn acts as a migratory source for Peru. However, seasonal winds in Ecuador during two periods of high fall armyworm levels are generally of low velocity and inconsistent direction (Fig 6). Such conditions raise the possibility that the fall armyworm populations in Ecuador could be relatively isolated from the rest of the continent.

Our findings of strong similarities in the COIB (Fig 5) and *Tpi* (Fig 6) haplotype compositions of fall armyworms from Ecuador, Peru, Bolivia, and Trinidad and Tobago are consistent with extensive mixing of the fall armyworm from these four locations, while those from Puerto Rico appear to be isolated. This result has important implications if true as Puerto Rico is only 100 km further from Ecuador than Trinidad and Tobago based on direct flight comparisons. It suggests that despite the known ability of fall armyworm to migrate thousands of kilometers per season in North America, the approximately 800 km of water separating Puerto Rico from the South American mainland (compared to only 50 km for Trinidad and Tobago) is a significant barrier preventing regular migration of fall armyworm from one site to the other.

To identify the most likely drivers of population movements in this region we examined two environmental factors that are known to significantly impact fall armyworm migration and dispersion patterns. These are wind vectors and the distribution of habitats with suitable climates. Substantial populations of fall armyworm were observed in cornfields in Ecuador during the spring and fall seasons. Wind vectors during these periods in most of Ecuador and Peru exhibit low velocity and inconsistent direction (Fig 6A), which are not compatible with the wind-dependent long-distance migration behavior observed in North America. This can be visualized by HYSPLIT analysis where we projected the direction and distance of dispersion promoted by wind vectors averaged for the months of March and September over a single 12-hour nocturnal flight period (Fig 6B). Dispersion projections from Trinidad and Tobago show a strong westward bias consistent with the strong prevailing wind vectors there, which contrasted with the more localized distributions originating from Ecuador and Peru.

**Fig 7:**
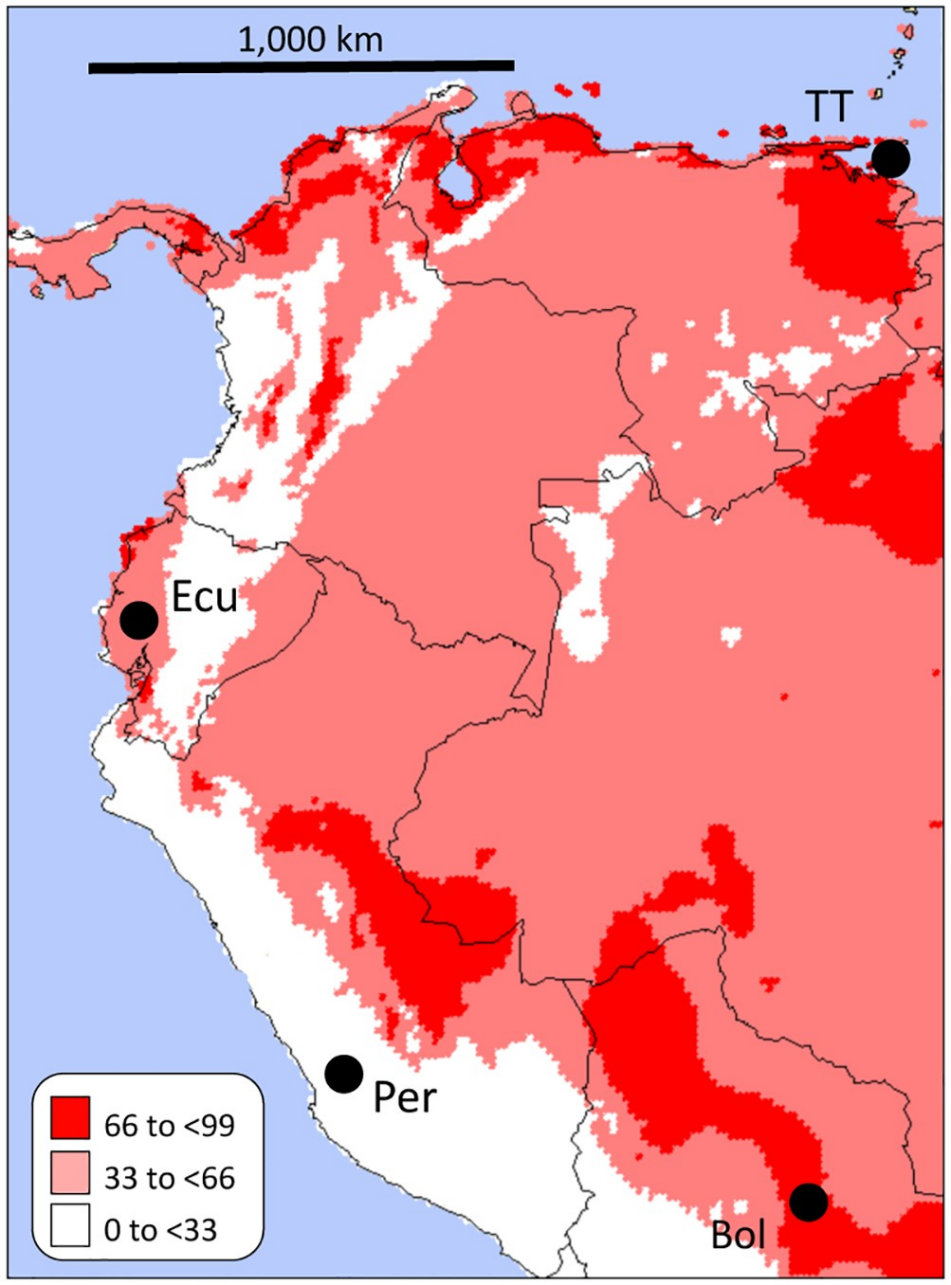
Map indicating the distribution of habitats in Ecuador and surrounding regions based on climate suitability for the development of fall armyworm. Map is based on CLIMEX calculations of the Establishment Index (EI) with higher values indicating more favorable climate.

More favorable to fall armyworm movements were the extensive areas with supportive habitats for fall armyworm populations (Fig 7). The CLIMEX analysis was set to show shading in map location with values equal or greater than 33, with an ecoclimatic index (EI) value of 30 or above considered highly favorable [42]. The EI climate suitability map describes a contiguous pathway of favorable habits from Trinidad and Tobago that extends through the eastern sections of Ecuador, Peru, and Bolivia. From these observations, if the widely dispersed fall armyworm from Trinidad and Tobago, Ecuador, Peru, and Bolivia represent a single interbreeding population as suggested by their genetic similarities, then movement between populations is likely to be dominated by short flights to adjacent favorable habitats rather than the long-distance wind-directed migration observed in North America. This migratory behavior should also be compatible with host plant availability as agricultural activity in the region, including corn production, largely overlap the areas of favorable climate for fall armyworm [43]. Less certain are the population densities that can be supported. The large migratory populations observed and modeled in North America are generally associated with extensive acreages of contiguous and high intensity corn plantings [4], conditions that tend to optimize population density and may not be typical of corn production in the northern region of South America. While speculative, these observations when considered in total suggests that fall armyworm movements in this region of South America may be associated with smaller migratory populations covering shorter distances in more variable directions.

In summary, it is important to note that these studies are limited to the fall armyworm infesting corn as collections from other host plants, particularly those associated with the rice-strain have been difficult to obtain in this region. Based on this sampling we conclude that the *COI* and *Tpi* compositions of fall armyworm found in corn in Ecuador are consistent with populations in the northern regions of South America extending from Trinidad and Tobago to the east, Peru to the south west, and Bolivia to the south undergoing substantial mixing due to migration and population dispersion. This pattern is consistent with the seasonal wind patterns consisting of strong eastward trade winds over Trinidad and Tobago that are deflected southward by the Andes mountain range in Peru as well as a favorable distribution of suitable habitats that could support localized movements of fall armyworm from Ecuador into eastern Peru and Bolivia. Evidence of substantial introgression of fall armyworm populations from Central America or the Greater Antilles into Ecuador have yet to be observed.

## Methods

### Specimen collections and DNA preparation

Ecuador specimens were obtained in 2018 as larvae from corn plants from two cantons in Manabi province, Portoviejo (March and September) and Tosagua (March) (Table 1). Collections from Peru, Puerto Rico, Trinidad and Tobago, Bolivia, multiple locations in Florida, and the Dominican Republic were described previously (Table 1). For these collections, the data for COIB were previously reported [33, 44], while data for *Tpi* were from this study. No endangered or protected species were involved in this study. We obtained permission from private farmers for access and data collection in their fields.

**Table 1:** Source information for fall armyworm collections.

Name	Country/State	Closest City	Coordinates		Collection Date
Ecu-Pv	Ecuador	Portoviejo	-1.07	-80.46	Mar, Sep 2018
Ecu-To	Ecuador	Tosagua	-0.78	-80.22	Sep 2018
TT^a^	Trinidad and Tobago	Port of Spain	10.65	-61.43	2013
Bol^a^	Bolivia	Cotoca	-17.77	-62.83	2012
Per^a^	Peru	Lima	-12.08	-76.93	2014
PR^b^	Puerto Rico	Isabela Juanita Diaz	18.50 18.06	-67.02 -66.51	2007, 2009 2009
DR^a^	Dominican Republic	Santo Domingo	18.51	-69.92	2015–2016
FL^a^	Florida	Miami	25.73	-80.38	2012–2014

^a^Collections described in [33].

^b^Collections described in [44].

Collected specimens were stored either air-dried or in ethanol at room temperature. A portion of each specimen was excised and homogenized in a 5-ml Dounce homogenizer (Thermo Fisher Scientific, Waltham, MA, USA) in 800 μl Genomic Lysis buffer (Zymo Research, Orange, CA, USA) and incubated at 55°C for 5–30 min. Debris was removed by centrifugation at 10,000 rpm for 5 min. The supernatant was transferred to a Zymo-Spin III column (Zymo Research, Orange, CA, USA) and processed according to manufacturer’s instructions. The DNA preparation was increased to a final volume of 100 μl with distilled water. Genomic DNA preparations of fall armyworm samples from previous studies were stored at -20°C. Species identity was initially determined by morphology and confirmed by sequence analysis of the COIB region.

### PCR amplification and DNA sequencing

PCR amplification for all segments was performed in a 30-μl reaction mix containing 3 μl 10X manufacturer’s reaction buffer, 1 μl 10mM dNTP, 0.5 μl 20-μM primer mix, 1 μl DNA template (between 0.05–0.5 μg), 0.5-unit Taq DNA polymerase (New England Biolabs, Beverly, MA). The thermocycling program was 94°C (1 min), followed by 33 cycles of 92°C (30 s), 56°C (45 s), 72°C (45 s), and a final segment of 72°C for 3 min. Typically 96 PCR amplifications were performed at the same time using either 0.2-ml tube strips or 96 well microtiter plates. All primers were obtained from Integrated DNA Technologies (Coralville, IA) and are mapped in Fig 1. Amplification of the COIB segment typically used the primer pair *924F* (5’-TTATTGCTGTACCAACAGGT-3’) and *1303R* (5’- CAGGATAGTCAGAATATCGACG-3’). When necessary nested PCR was used in which the first PCR was performed using primers *891F* (5’-TACACGAGCATATTTTACATC-3’) and *1472R* (5’-GCTGGTGGTAAATTTTGATATC-3’) followed by a second PCR using the internal primers *924F* and *1303R*. Amplification of the *Tpi* exon-intron segment used the primers *412F* (5’- CCGGACTGAAGGTTATCGCTTG -3’) and *1140R* (5’- GCGGAAGCATTCGCTGACAACC-3’) to produce a variable length fragment due to insertion and deletion mutations. Nested PCR was again used when needed with the first PCR done with primers *634F* (5’-TTGCCCATGCTCTTGAGTCC-3’) and *1166R* (5’-TGGATACGGACAGCGTTAGC-3’) and the second PCR using the internal primers *412F* and *1140R*.

For fragment isolations, 6 μl of 6X gel loading buffer was added to each amplification reaction and the entire sample run on a 1.8% agarose horizontal gel containing GelRed (Biotium, Hayward, CA) in 0.5X Tris-borate buffer (TBE, 45 mM Tris base, 45 mM boric acid, 1 mM EDTA pH 8.0). Fragments were visualized on a long-wave UV light box and manually cut out from the gel. Fragment isolation was performed using Zymo-Spin I columns (Zymo Research, Orange, CA) according to manufacturer’s instructions. The University of Florida Interdisciplinary Center for Biotechnology (Gainesville, FL) and Genewiz (South Plainfield, NJ) performed the DNA sequencing.

DNA alignments and consensus building were performed using MUSCLE (multiple sequence comparison by log-expectation), a public domain multiple alignment software incorporated into the Geneious Pro 10.1.2 program (Biomatters, New Zealand, http://www.geneious.com) [45]. Phylogenetic trees were graphically displayed in a neighbor-joining (NJ) tree analysis also included in the Geneious Pro 10.1.2 program [46]. Phylogenetic networks were estimated by the TCS statistical parsimony algorithm ([47]) incorporated in the software program PopArt [48].

### Characterization of the *CO1* and *Tpi* gene segments

The genetic markers are all single nucleotide substitutions. Sites in the *COI* gene are designated by an "m" (mitochondria) while *Tpi* sites are designated "g" (genomic). This is followed by the DNA name, number of base pairs from the predicted translational start site (*COI*), 5’ start of exon (*Tpi*), or 5’ start of the intron (TpI4) and the nucleotides observed using IUPAC convention (R: A or G, Y: C or T, W: A or T, K: G or T, S: C or G, D: A or G or T).

The *COI* markers are from the maternally inherited mitochondrial genome. The COIB segment was amplified by *CO1* primers 891F and 1472R (Fig 1A). Overlapping COIB segments for various *Spodoptera* species were obtained from GenBank and included, *S*. *abula* (HQ177287), *S*. *cosmiodes* (HQ177295), *S*. *descoinsi* (HQ177306), *S*. *dolichos* (HQ177313), *S*. *eridania* (Stoll in Cramer)(HQ177321), *S*. *exempta* (Walker)(HQ177334), *S*. *exigua* (Hübner)(HQ177339), *S*. *latisfscia* (Walker)(HQ177354), *S*. *littoralis* (Boisduval)(HQ177364), *S*. *mauritia* (Boisduval)(HQ177382), *S*. *ornithogalli* (Guenée)(HQ177392), *S*. *praefica* (Grote)(HQ177407), *S*. *litura* (F.)(HQ177375). These sequences had in common a 259-bp segment designated COIB259, which was used for species identification by phylogenetic analysis.

The larger COIB296 segment was used for all other COI analyses (Fig 1A). Sites mCOI1164D and mCOI1287R are diagnostic for strain identity in Western Hemisphere populations where there is a single rice-strain, T_1164_A_1287_, and four corn-strain configurations, A_1164_A_1287_ (h1), A_1164_G_1287_ (h2), G_1164_A_1287_ (h3), G_1164_G_1287_ (h4) [33].

Variants in the *Tpi* e4 exon segment can also be used to identify host strain identity with results generally comparable with the *CO1* marker [28]. The gTpi183Y site is on the fourth exon of the predicted *Tpi* coding region and was PCR amplified using the *Tpi* primers 412F and 1140R (Fig 1B). The C-strain allele (*Tpi*C) is indicated by a C_183_ and the R-strain (*Tpi*R) by T183 [28]. The *Tpi* gene is located on the *Z* sex chromosome that is present in one copy in females and two copies in males. Because the genomic DNA was directly sequenced, males heterozygous for *Tpi* alleles will simultaneously display both alternatives at polymorphic sites, which if different are easily identified by overlapping sequencing chromatographs. Heterozygosity at site *Tpi*_183_ was limited to C/T and was denoted as *Tpi*H.

The Tpie4i4 segment includes a 52 bp portion of the e4 exon followed by approximately 172 bp of the TpI4 intron, the latter of which is of variable length due to frequent insertions and deletions (indels). The segment was sequenced with primer 891F for the initial sequencing reaction and 1140R for 2^nd^ strand sequence confirmation when needed in cases of ambiguity. This segment was chosen for analysis because it empirically had the most consistent sequence quality with the given primers. A variable but often high percentage of specimens were heterozygous for frameshift mutations in the intron that could be identified by overlapping chromatographs immediately after the polymorphism. These were not further analyzed.

### Calculation of haplotype numbers

The mitochondrial *COI* markers are calculated directly as the number of specimens exhibiting the *COI* haplotypes divided by total specimens. Because *Tpi* is a sex-linked nuclear gene, the number of *Tpi* genes present will differ by sex. Specimens with a TpiC or TpiR haplotype could be either a homozygous male or hemizygous female. We assumed a 1:1 sex ratio for the larval specimens so that the average number of *Tpi* genes per specimen is given as 1.5 as calculated: (2 in males + 1 in females)/2. Based on this reasoning the number of TpiC and TpiR specimens were multiplied by 1.5 to estimate the number of chromosomes carrying each marker. In comparison, TpiH specimens are heterozygous and so carried one of each marker. The estimated number of TpiC chromosomes was calculated by 1.5TpiC + TpiH, and TpiR chromosomes = 1.5TpiR + TpiH.

### CLIMEX climate suitability analysis

CLIMEX is a dynamic simulation model that estimates the potential geographical distribution and relative abundance of a species according to climate [42]. In principle, extrapolating locations with favorable climate based on the biological parameters of a species should provide a reasonable estimate of geographical distribution, and for pests identify locations at high risk of infestation. CLIMEX projections for fall armyworm has been performed at continental and global scales and while there were a few variations in the biological parameters for fall armyworm used between studies these generated only modest differences in the distribution maps [49–51]. We performed a CLIMEX suitability analysis focused on the northern portion of South America, using fall armyworm parameters from du Plessis et al (2018) [49] (Table 2). Climate information was imported from Climond (www.climond.org) [52], using historical data from 1961–1990 at a resolution of 10’.

**Table 2:** CLIMEX parameter values used for modelling fall armyworm [46].

Parameter	Description	Value
Moisture		
SM0	Lower soil moisture threshold	0.15
SM1	Lower optimal soil moisture	0.8
SM2	Upper optimal soil moisture	1.5
SM3	Upper soil moisture threshold	2
.5Temperature		
DV0	Lower temperature threshold	12°C
DV1	Lower optimal temperature	25°C
DV2	Upper optimal temperature	30°C
DV3	Upper temperature threshold	39°C
Cold Stress		
TTCS	Cold stress temperature threshold	12°C
THCS	Cold stress accumulation rate	-0.001 week^-1^
Heat Stress		
TTHS	Heat stress temperature threshold	39°C
THHS	Heat stress accumulation rate	0.005 week^-1^
Dry Stress		
SMDS	Soil moisture dry stress threshold	0.1
HDS	Dry stress accumulation rate	-0.005 week^-1^
Wet Stress		
SMWS	Soil moisture wet stress threshold	3
HWS	Wet stress accumulation rate	0.002 week^-1^
Minimum degree-day sum needed to complete a generation		
PDD	Degree-Days per generation	600°C

The Ecoclimatic Index (EI) is calculate from the annual Growth Index (GI) and the Stress Index (SI). The GI combines the minimum limit, optimum lower, optimum upper, and maximum limit for fall armyworm temperature and moisture indices calculated on a weekly basis, then averaged for the year to provide a measure of population growth potential. This is counterbalanced by the SI, which is a measure of unfavorable conditions focused primarily on temperature and precipitation. The EI integrates the GI and SI and is presented on a 0–100 scale, where 100 represents 100% suitability throughout the year (as would occur in an incubator). EI values greater than 30 are considered to be favorable for the long-term survival of the species in the region [42].

For this study, the Compare Locations (1 species) function in the CLIMEX program was used with the Grid Data simulation file. The species’ known distribution data was imported from CABI’s Invasive Species Compendium (www.cabi.org/isc). The location component was set to CM:10 South America. No climate change scenario or irrigation components were set. An EI map was created from the simulation, with the map specifications set at 0.16 diameter circles, showing zeros with three legend items.

### HYSPLIT air trajectory projections

Air transport trajectories for select locations were estimated using the Hybrid Single Particle Lagrangian Integrated Trajectory Model at the Air Resources Laboratory (ARL) READY web site run by NOAA (http://ready.arl.noaa.gov/HYSPLIT.php) [53]. Projections were made for air transport conditions averaged for the 30-day period from March 1–30, 2018 and September 1–30, 2018. Fall armyworm migrates nocturnally so the duration of continuous flight was limited to a 12-hour period beginning at dusk, with a starting altitude of 500 m AGL and a maximum altitude of 1500 m AGL [4]. The pathways of the projections from each location were averaged and displayed as a frequency distribution with percentages reflecting the proportion of trajectories entering a given grid.

## Supporting information

S1 Fig Consensus sequences for *COI*-CS and *COI*-RS. (TIF)
