# Supplementary figures and images for: Genetic characterization of fall armyworm (Spodoptera frugiperda) in Ecuador and comparisons with regional populations identify likely migratory relationships
Source: PLoS One. 2019 Sep 19;14(9):e0222332. doi: 10.1371/journal.pone.0222332 (PMC6752802; doi:10.1371/journal.pone.0222332)

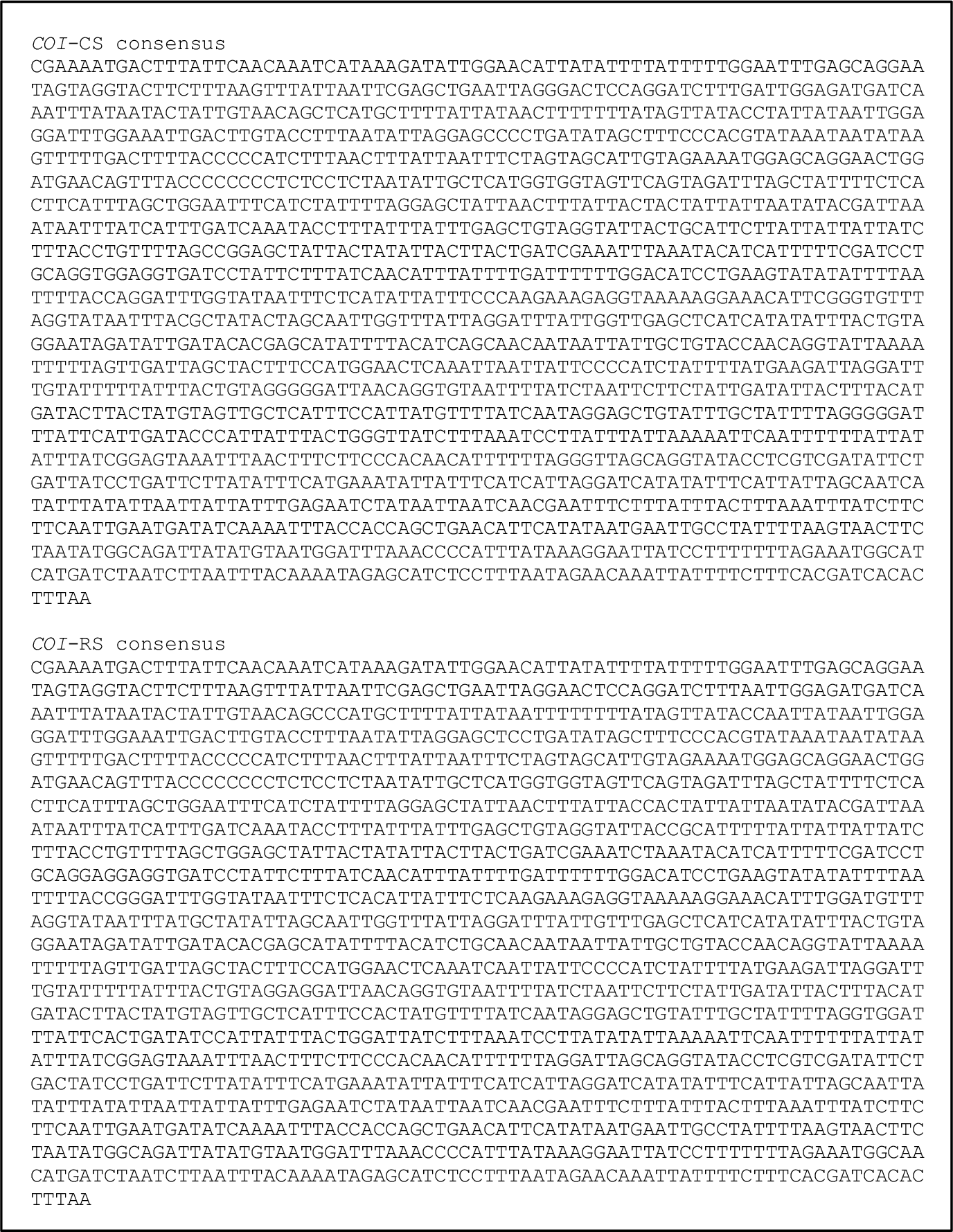

Supplement: S1 Fig — (TIF) [file pone.0222332.s001.tif]
